# Supplementary material for: Machine Learning Accelerates Crystallization for Structure Determination
Source: Angew Chem Int Ed Engl. 2026 May 4;65(25):e1218503. doi: 10.1002/anie.1218503 (PMC13266958; doi:10.1002/anie.1218503)

# checkCIF/PLATON report

Structure factors have been supplied for datablock(s) lcz061_1

THIS REPORT IS FOR GUIDANCE ONLY. IF USED AS PART OF A REVIEW PROCEDURE FOR PUBLICATION, IT SHOULD NOT REPLACE THE EXPERTISE OF AN EXPERIENCED CRYSTALLOGRAPHIC REFEREE.

No syntax errors found. CIF dictionary Interpreting this report

# Datablock: lcz061_1

---

```
Bond precision:   C-C = 0.0155 A          Wavelength=1.54184

Cell:               a=8.5596(1)       b=9.4113(1)       c=56.4718(6)
                    alpha=90          beta=90           gamma=90
Temperature:        100 K
```

| | Calculated | Reported |
|---|---|---|
| Volume | 4549.20(9) | 4549.20(9) |
| Space group | P 21 21 21 | P 21 21 21 |
| Hall group | P 2ac 2ab | P 2ac 2ab |
| Moiety formula | C30 H15 Ag3 F18 N6 O2, C15 H12 O2 | C15 H3 Ag3 F18 N6, 2(C15 H12 O2) |
| Sum formula | C45 H27 Ag3 F18 N6 O4 | C45 H27 Ag3 F18 N6 O4 |
| Mr | 1381.34 | 1381.33 |
| Dx,g cm-3 | 2.017 | 2.017 |
| Z | 4 | 4 |
| Mu (mm-1) | 11.420 | 11.420 |
| F000 | 2696.0 | 2696.0 |
| F000’ | 2707.86 | |
| h,k,lmax | 10,11,71 | 10,11,69 |
| Nref | 9693[ 5510] | 8879 |
| Tmin,Tmax | 0.167,0.227 | 0.452,1.000 |
| Tmin’ | 0.038 | |

Correction method= # Reported T Limits: Tmin=0.452 Tmax=1.000
AbsCorr = MULTI-SCAN

Data completeness= 1.61/0.92        Theta(max)= 77.785

R(reflections)= 0.0531( 8344)        wR2(reflections)= 0.1256( 8879)

S = 1.068        Npar= 688

---

The following ALERTS were generated. Each ALERT has the format
**test-name_ALERT_alert-type_alert-level.**
Click on the hyperlinks for more details of the test.

---

### Alert level C

```
PLAT042_ALERT_1_C Calc. and Reported MoietyFormula Strings  Differ     Please Check
              Calc: C30 H15 Ag3 F18 N6 O2, C15 H12 O2
              Rep.: C15 H3 Ag3 F18 N6, 2(C15 H12 O2)
PLAT090_ALERT_3_C Poor Data / Parameter Ratio (Zmax > 18) ........       7.82 Note
PLAT094_ALERT_2_C Ratio of Maximum / Minimum Residual Density ....       2.14 Report
PLAT234_ALERT_4_C Large Hirshfeld Difference O00F     --C020     .       0.16 Ang.
PLAT342_ALERT_3_C Low Bond Precision on  C-C Bonds ...............     0.0155 Ang.
PLAT906_ALERT_3_C Large K Value in the Analysis of Variance ......      2.077 Check
PLAT911_ALERT_3_C Missing FCF Refl Between Thmin & STh/L=    0.600          2 Report
                 8  6 14,   9  0 30,
PLAT971_ALERT_2_C Check Calcd Resid. Dens.  1.47Ang From C00W            2.40 eA-3
PLAT971_ALERT_2_C Check Calcd Resid. Dens.  1.51Ang From O00F            2.21 eA-3
PLAT975_ALERT_2_C Check Calcd Resid. Dens.  1.09Ang From O00F    .       0.68 eA-3
PLAT977_ALERT_2_C Check Negative Difference Density on H01N      .      -0.36 eA-3
```

---

### Alert level G

```
PLAT007_ALERT_5_G Number of Unrefined Donor-H Atoms .............          2 Report
              H00H  H00G
PLAT033_ALERT_4_G Flack x Value Deviates > 3.0 * Sigma from Zero .      0.175 Note
PLAT083_ALERT_2_G SHELXL Second Parameter in WGHT  Unusually Large      28.24 Why ?
PLAT242_ALERT_2_G Low    'MainMol' Ueq as Compared to Neighbors of      C01D Check
PLAT242_ALERT_2_G Low    'MainMol' Ueq as Compared to Neighbors of      C01L Check
PLAT242_ALERT_2_G Low    'MainMol' Ueq as Compared to Neighbors of      C01T Check
PLAT242_ALERT_2_G Low    'MainMol' Ueq as Compared to Neighbors of      C01V Check
PLAT242_ALERT_2_G Low    'MainMol' Ueq as Compared to Neighbors of      C01Z Check
PLAT242_ALERT_2_G Low    'MainMol' Ueq as Compared to Neighbors of      C021 Check
PLAT720_ALERT_4_G Number of Unusual/Non-Standard Labels ..........        103 Note
              Ag01    Ag02    Ag03    F004    F005    F006    F007    F008
              F009    F00A    F00B    F00C    F00D    F00E    O00F    O00G
              H00G    O00H    H00H    F00I    O00J    F00K    F00L    N00M
              F00N    F00O    N00P    F00Q    N00R    F00S    N00T    N00U
              N00V    C00W    C00X    C00Y    H00Y    C00Z    C010    C011
              C012    H012    C013    C014    C015    H015    C016    C017
              H017    C018    H018    C019    C01A    H01A    H01B    C01B
              C01C    H01C    C01D    C01E    H01E    C01F    H01D    H01F
              C01G    H01G    C01H    C01I    C01J    C01K    C01L    C01M
              H01M    C01N    H01N    C01O    H01O    C01P    H01P    C01Q
              H01H    H01I    C01R    H01R    C01S    H01S    C01T    C01U
              H01U    C01V    C01W    C01X    H01X    C01Y    C01Z    C020
              C021    C022    H022    C023    C024    H02A    H02B
PLAT790_ALERT_4_G Centre of Gravity not Within Unit-Cell: Resd.  #          2 Note
```

```
             C15 H12 O2
PLAT910_ALERT_3_G Missing FCF Reflection(s) Below Theta(Min)[Deg]=       3.13 Note
                0  0  2,
PLAT912_ALERT_4_G Missing # of FCF Reflections Above STh/L=  0.600        113 Note
PLAT941_ALERT_3_G Average HKL Measurement Multiplicity ..........        3.9 Low
PLAT952_ALERT_5_G Calculated (ThMax) and CIF-Reported Lmax Differ.          2 Units
PLAT958_ALERT_1_G Calculated (ThMax) and Actual (FCF) Lmax Differ.          2 Units
PLAT969_ALERT_5_G The 'Henn et al.' R-Factor-gap value ..........      2.025 Note
             Predicted wR2: Based on SigI**2  6.20 or SHELX Weight 11.76
PLAT978_ALERT_2_G Number C-C Bonds with Positive Residual Density.          0 Info
```

---

```
   0 ALERT level A = Most likely a serious problem - resolve or explain
   0 ALERT level B = A potentially serious problem, consider carefully
  11 ALERT level C = Check. Ensure it is not caused by an omission or oversight
  18 ALERT level G = General information/check it is not something unexpected

   2 ALERT type 1 CIF construction/syntax error, inconsistent or missing data
  13 ALERT type 2 Indicator that the structure model may be wrong or deficient
   6 ALERT type 3 Indicator that the structure quality may be low
   5 ALERT type 4 Improvement, methodology, query or suggestion
   3 ALERT type 5 Informative message, check
```

---

## Validation response form

Please find below a validation response form (VRF) that can be filled in and pasted into your CIF.

```
# start Validation Reply Form
_vrf_PLAT042_lcz061_1
;
PROBLEM: Calc. and Reported MoietyFormula Strings  Differ     Please Check
RESPONSE: ...
;
_vrf_PLAT090_lcz061_1
;
PROBLEM: Poor Data / Parameter Ratio (Zmax > 18) ........       7.82 Note
RESPONSE: ...
;
_vrf_PLAT094_lcz061_1
;
PROBLEM: Ratio of Maximum / Minimum Residual Density ....       2.14 Report
RESPONSE: ...
;
_vrf_PLAT234_lcz061_1
;
PROBLEM: Large Hirshfeld Difference O00F     --C020     .       0.16 Ang.
RESPONSE: ...
;
_vrf_PLAT342_lcz061_1
;
PROBLEM: Low Bond Precision on  C-C Bonds ..............     0.0155 Ang.
RESPONSE: ...
;
_vrf_PLAT906_lcz061_1
;
PROBLEM: Large K Value in the Analysis of Variance ......      2.077 Check
```

```
RESPONSE: ...
;
_vrf_PLAT911_lcz061_1
;
PROBLEM: Missing FCF Refl Between Thmin & STh/L=    0.600          2 Report
RESPONSE: ...
;
_vrf_PLAT971_lcz061_1
;
PROBLEM: Check Calcd Resid. Dens.  1.47Ang From C00W           2.40 eA-3
RESPONSE: ...
;
_vrf_PLAT975_lcz061_1
;
PROBLEM: Check Calcd Resid. Dens.  1.09Ang From O00F    .       0.68 eA-3
RESPONSE: ...
;
_vrf_PLAT977_lcz061_1
;
PROBLEM: Check Negative Difference Density on H01N      .      -0.36 eA-3
RESPONSE: ...
;
# end Validation Reply Form
```

---

It is advisable to attempt to resolve as many as possible of the alerts in all categories. Often the minor alerts point to easily fixed oversights, errors and omissions in your CIF or refinement strategy, so attention to these fine details can be worthwhile. It is up to the individual to critically assess their own results and, if necessary, seek expert advice.

---

**PLATON version of 17/09/2025; check.def file version of 17/09/2025**

---

# duplicate check

**No duplication found**

---

**Datablock lcz061_1** - ellipsoid plot

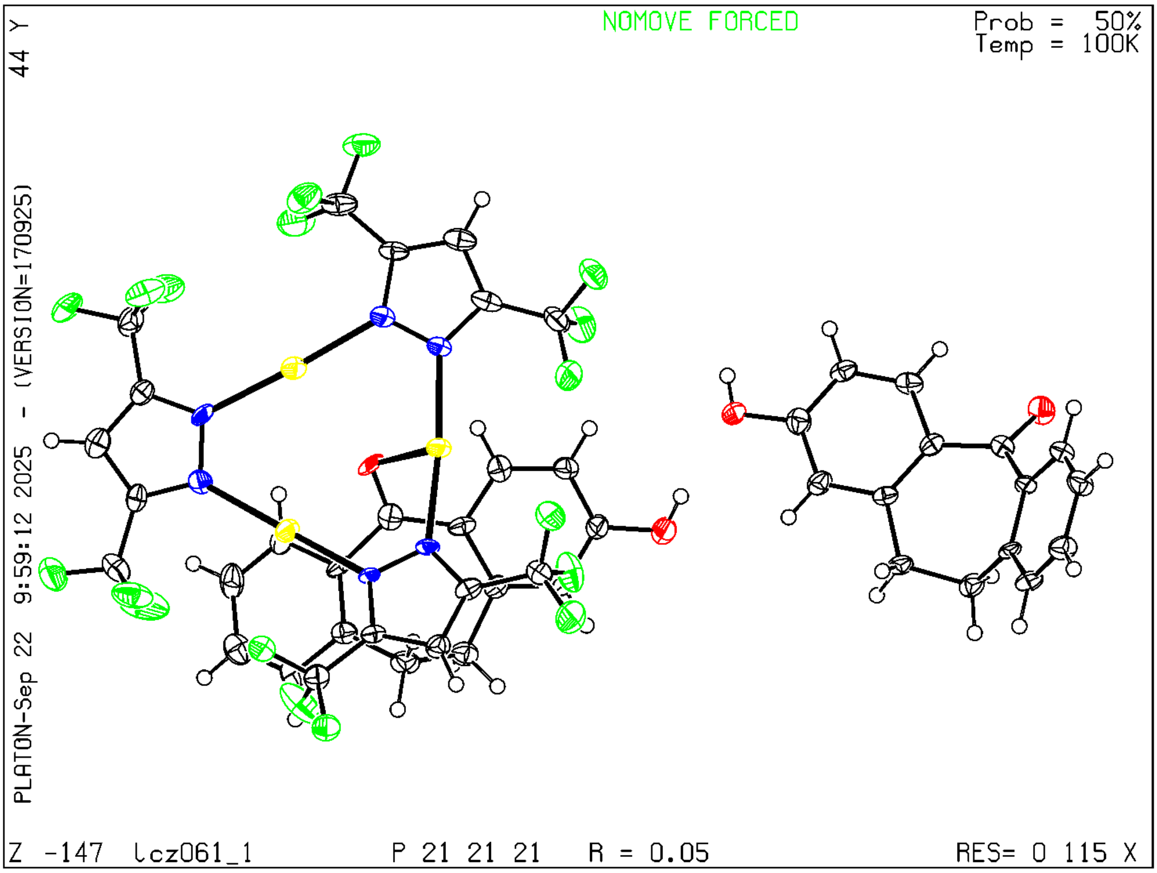

Supplement: Supplementary file 2 — Supporting File 2: anie72462‐sup‐0002‐DataFile.zip. [file ANIE-65-e1218503-s002.zip › supplementary_materials_crystallographic_data submit.zip!/cif_and_checkcif(1-114)/Ag3Pz3_61/Ag3Pz3_61_checkcif.pdf]
